# Secondary vs. Primary Spinal Infection in Early Clinical Assessment: A Parsimonious, Leakage-Resistant Modelling Approach with Internal Validation: A Multicenter Retrospective Study

**DOI:** 10.3390/jcm15051873

**Published:** 2026-02-28

**Authors:** Merih Can Yilmaz, Ozgur Ozaydin, Cengiz Cokluk, Keramettin Aydin

**Affiliations:** 1 Department of Neurosurgery, VM Medical Park Hospital, 55200 Samsun, Turkey; 2 Department of Economics, Ondokuz Mayis University, 55270 Samsun, Turkey; ozaydin@omu.edu.tr; 3 Department of Neurosurgery, Faculty of Medicine, Ondokuz Mayis University, 55270 Samsun, Turkey; cengizcokluk@gmail.com

**Keywords:** spinal infection, chronic kidney disease, diabetes mellitus, exploratory modeling

## Abstract

**Background and Objectives**: Spinal infections represent a heterogeneous group of diseases where primary or secondary etiological classification is fundamental for diagnosis and clinical decision-making. The aim is to present multicenter data evaluating etiological patterns associated with comorbidity. This study investigated the etiological distribution of spinal infections in a multicenter cohort and examined the relationships between chronic kidney disease (CKD) and diabetes mellitus (DM) and primary and secondary spinal infection etiologies, which emerged in the study and are thought to contribute to the literature. **Materials and Methods**: For this early-phase exploratory modelling study, a ridge-penalized logistic regression (L2) model was trained using repeated nested cross-validation (outer 5-fold stratified CV ×10 repetitions; inner 5-fold CV) to generate out-of-fold (OOF) probabilities. The penalty parameter (C) was optimized by minimizing log-loss. All preprocessing was performed within the CV pipeline to prevent data leakage. A supplementary Firth-penalized analysis was conducted as a plausibility check, using the CKD0/DM0 group as reference. **Results**: The model demonstrated effective discrimination between spinal infection probabilistic profiles (OOF AUC 0.762; conditional OOF bootstrap 95% CI 0.608–0.885). A contrasting probabilistic profile concordance was observed: DM-only patients had a high likelihood of secondary infection (observed secondary risk 93.3%; mean OOF estimated probability 84.4%), compared to a higher likelihood of primary infection in CKD-only patients (observed secondary risk 15.4%, which translates to a primary risk of 84.6%; mean OOF estimated probability 21.8%). Calibration was near-ideal (intercept 0.069; slope 1.028). Decision curve analysis showed a clear utility between the thresholds of 0.15 and 0.84. There were no CKD+DM+ cases (*n* = 0); analyses were restricted to supported strata. **Conclusions**: In this multicenter analysis of spine infections, CKD was predominantly related to primary spine infection etiology, whereas DM was more frequently related to secondary spine infections. These findings emphasize the potential role of comorbidity profiles in etiologic classification and need to be confirmed in larger multicenter cohorts.

## 1. Introduction

Spinal infections are serious infectious diseases affecting the spine and surrounding structures. They usually affect the vertebral bodies, vertebral discs, epidural space and soft tissues around the spine and account for 2–7% of all musculoskeletal infections [1]. The clinical course is typically characterized by pain, fever and neurologic disturbances, and the diagnosis is often delayed. Patients with spinal infections who show thoracic involvement, limb weakness, bladder dysfunction, urinary problems or cauda equina syndrome are at higher risk of a poor prognosis [2]. This raises the risk of permanent neurological damage and disease.

Most of the patients diagnosed with spinal infection can be effectively treated in the early stages with conservative methods such as antibiotic therapy, bed rest and spinal immobilization.

Surgical intervention requires significant or impending spinal instability, worsening neurologic impairment, formation of a spinal abscess, failure to respond to conservative treatment, severe clinical signs suggestive of sepsis, or recurrence after initial non-surgical treatment. Microbiologic samples can be obtained by percutaneous computed tomography-guided biopsy or open surgical biopsy. Whenever possible, specimen collection should be performed before starting antibiotic therapy [3,4]. Whatever the treatment approach, individuals with spinal infection need careful follow-up, including repeated neurological assessments and imaging to monitor disease progression or recovery [4].

Spinal infections can be etiologically classified as primary and secondary. Primary spinal infections are usually caused by hematogenous spread from a distant focus of infection to the spinal column. Secondary spinal infections often occur because of surgical intervention, spinal trauma or direct spread from adjacent tissues. Studies indicate that chronic renal failure and diabetes mellitus are predisposing factors in spinal infections [5,6,7,8,9]. However, these etiological factors have not been studied in the early diagnostic phase, nor have they been modeled within an explanatory framework, for differentiating between primary and secondary spinal infections.

Clinical findings are often non-specific in the diagnostic process; therefore, laboratory tests and advanced imaging modalities, especially magnetic resonance imaging (MRI), which is the modality with the best sensitivity and specificity for spinal infection, are of great importance [10,11]. Positron emission tomography (PET) using FDG demonstrates high sensitivity in identifying spinal infections, distinguishing between various subtypes, and detecting the extension of infection beyond the spinal column. [18F] FDG PET/CT is particularly valuable in cases where MRI results are inconclusive, when MRI is contraindicated, or in patients with bacteremia where it aids in localizing infectious foci [12]. Accurate differential diagnosis of primary and secondary infections plays a critical role in determining the appropriate treatment strategy. However, culture negativity in secondary infections remains a common challenge in clinical practice [13].

Advances in imaging technology, pharmacologic therapies and surgical techniques have significantly improved clinical outcomes and markedly reduced morbidity and mortality associated with spine infections. Effective management now requires a collaborative, multidisciplinary approach involving specialists in infectious diseases, neuroradiology and spine surgery. Timely and accurate diagnosis continues to be the cornerstone of successful treatment in these cases [14].

Spinal infections present a clinically heterogeneous condition and, in this setting, timely differentiation between secondary and primary etiology can shape the direction of diagnosis, the urgency and extent of resource search, imaging strategy and microbiological sampling plans. In patients with chronic kidney disease and/or diabetes, the clinical context often elicits conflicting hypotheses: secondary spinal infection may require intensification of the resource search and early multidisciplinary planning, whereas primary spinal infection may prioritize evaluation of hematogenous spread and careful longitudinal monitoring. As both etiologies have clinically important consequences, a balanced task of differentiation is required, treating secondary and primary infection as equally meaningful targets.

Solid clinical predictive modeling often benefits from large cohorts and external validation. The primary aim of this study was to make an etiologic distinction, distinguish secondary spinal infection from primary spinal infection, and assess whether chronic kidney disease (CKD) and diabetes (DM) are compatible with different infection type profiles. Early-phase datasets can still support this aim when established best practices are applied, particularly strict leakage prevention, appropriate penalization, and transparent internal validation. Accordingly, we apply an established ridge-penalized modeling strategy with strict leakage precautions to obtain realistic internal performance estimates and to characterize opposite-direction probabilistic profile alignment.

To differentiate secondary versus primary spinal infection while minimizing common pitfalls in early-phase explanatory model (optimism, leakage, and unstable probability estimates), we adopted a TRIPOD-consistent two-track strategy with strict leakage precautions. First, we develop a ridge-penalized logistic regression model evaluated by repeated nested cross-validation, reporting discrimination, calibration, overall performance, and decision-analytic utility. Second, we provide an association-oriented plausibility summary using Firth penalization to contextualize CKD/DM contrasts as descriptive (non-causal) evidence rather than confirmatory causal inference. Our central hypothesis specifies opposite-direction probabilistic profile alignment: DM aligns with the secondary probabilistic profile, while CKD aligns with the primary probabilistic profile, within the supported strata of the dataset.

### Contributions

- We frame etiologic discrimination of spinal infection type (secondary vs. primary) as a balanced two-probabilistic profile task in patients with CKD and/or DM.
- We apply an established ridge-penalized logistic regression strategy with repeated nested cross-validation (and with standard leakage precautions) to obtain patient-level OOF probabilities and internal performance estimates.
- We report discrimination, calibration, decision-analytic utility, and support-aware subgroup profiling with explicit Figure/Table-linked presentation.
- We provide a supplementary Firth-penalized plausibility summary (Supplementary Table S1) to contextualize CKD/DM contrasts.

## 2. Materials and Methods

This retrospective multicenter study was conducted using data obtained from two tertiary referral centers. Medical records of patients diagnosed with spinal infections over 10 years were reviewed. A total of 52 patients meeting the inclusion criteria were identified. The cohort included 29 males and 23 females, with a mean age of 60.4 years. Demographic data, clinical presentation, radiological findings, and microbiological results were analyzed. Spinal infections were classified as primary or secondary based on etiology. Primary infections were defined as hematogenous infections without an identifiable source, while secondary infections were defined as infections associated with previous spinal surgery, invasive procedures, or spread from adjacent structures; for example, psoas abscess-associated cases were classified as secondary. Ethical approval was obtained from the Institutional Ethics Committee of Ondokuz Mayıs University Faculty of Medicine Hospital, application number 2025/186, approval date 25 April 2025.

### 2.1. Study Design and Reporting Approach

To distinguish between secondary and primary spinal infection probabilistic profiles, we conducted a model development and internal validation study following a TRIPOD-compliant structure emphasizing transparent model specification, leakage prevention, validation design, performance evaluation (discrimination, calibration, overall error), and decision analytic assessment.

### 2.2. Data Source and Participants

The dataset includes *n* = 52 patient records with documented type of spinal infection (secondary vs. primary) and recorded chronic kidney disease (CKD) and diabetes (DM) status; this includes patients with CKD and/or DM as well as patients with neither of these conditions (CKD0/DM0). All records were used for model development and internal validation.

### 2.3. Missing Data

There were no missing data on outcome or predictor variables; all 52 records were included in the analysis (0 exclusions; no missing data completion).

### 2.4. Outcome Definition

Binary outcome was the type of spinal infection, coded as secondary infection and primary infection. The positive class was defined as infectiontype = 1 (secondary spinal infection), and infectiontype = 0 denotes primary spinal infection.

### 2.5. Index Time and Temporality

All predictors (sex, age, CKD, and DM) were defined and available at index time (initial clinical assessment/admission). The spinal infection type classification (secondary vs. primary) was ascertained after index time within the predefined clinical observation window. This ordering ensured that predictor information did not post-date the outcome definition, preventing target leakage.

### 2.6. Predictors and Handling of Predictors

Predictors were prespecified based on clinical plausibility and availability at index time: sex (binary; 0/1 as recorded), age (continuous), CKD (binary; 0/1), and DM (binary; 0/1).

Age was modeled as a linear term on the logit scale to preserve parsimony and estimation stability in this early-phase setting. Assessment of potential non-linear age effects (e.g., splines or fractional polynomials) is a natural next step for larger cohorts and is not required to establish the present probabilistic profile-alignment signal.

### 2.7. Support-Aware Strata

The dataset contained no observations with concurrent CKD = 1 and DM = 1 (CKD+DM+; *n* = 0). Therefore, model interpretation and subgroup reporting were restricted to CKD–DM strata supported by the data, and any use of the model for CKD+DM+ patients should be considered extrapolative pending validation in cohorts that include this subgroup.

### 2.8. Model Specification: Explanatory Analysis Track

We used ridge-penalized logistic regression (L2) to obtain stable probability estimates and to mitigate coefficient instability and overly extreme predicted probabilities that can arise in unpenalized logistic regression under limited information. Predictors were standardized within the modeling pipeline to place them on comparable scales for penalization. The ridge classifier was implemented as L2-penalized logistic regression using a liblinear solver with a maximum of 5000 iterations; all predictors were standardized within the pipeline (mean-centering and scaling to unit variance) based only on the training folds.

### 2.9. Internal Validation: Repeated Nested Cross-Validation

The analysis was implemented in Python (v3.13.9) using scikit-learn (ridge logistic regression and nested CV, v1.8.0), statsmodels (calibration intercept/slope, v0.14.6), and NumPy (v2.4.0)/Pandas (v2.3.3)/Matplotlib (data handling and figures, v3.10.8). Age was modeled as a linear continuous predictor (age_con); non-linear age effects were not explored.

To tune hyperparameters and generate out-of-fold (OOF) probabilities for every patient, we used repeated nested cross-validation. The outer loop used 5-fold stratified cross-validation repeated 10 times (50 outer fits total) to generate repeat-specific OOF probabilities for each patient. The inner loop used 5-fold stratified cross-validation within each outer training set to tune the ridge hyperparameter C (the reciprocal of penalty strength) over a prespecified grid, optimizing log-loss to prioritize accurate risk probabilities.

Implementation details (reproducibility): The outer loop used RepeatedStratifiedKFold (5 splits, 10 repeats; random_state = 42) to ensure each patient received 10 out-of-fold risk scores. Within each outer training set, hyperparameter tuning used StratifiedKFold (5 splits, shuffle = True; random_state = 42) inside GridSearchCV, selecting C by minimizing log-loss (scoring = neg_log_loss). The C grid comprised 21 logarithmically spaced values from 10^−4^ to 10^4^ (numpy.logspace(−4, 4, 21)).

### 2.10. Aggregation of Repeated OOF Probabilities

Each outer repeat yields exactly one OOF probability per patient; thus, each patient receives 10 repeat-specific OOF probabilities. We computed a single patient-level OOF probability as the arithmetic mean across the 10 repeat-specific OOF probabilities. All reported performance metrics, plots, and uncertainty summaries were computed using these single-valued patient-level OOF probabilities.

### 2.11. Performance Assessment

Using the single-valued patient-level OOF probabilities, we evaluated discrimination (AUC), overall performance (Brier score, log-loss), calibration (calibration intercept and slope via logistic recalibration), and decision-analytic utility via decision curve analysis (DCA, which was included as a methodological illustration of potential net-benefit behavior and should not be interpreted as evidence of current clinical utility). For the calibration plot, we used 10 quantile-based bins (strategy = “quantile”). For DCA, net benefit was evaluated over threshold probabilities from 0.01 to 0.99 in 99 equally spaced steps.

### 2.12. Uncertainty Quantification (Conditional OOF Bootstrap)

We summarized uncertainty using a nonparametric bootstrap with 3000 resamples applied to paired (outcome, single-valued OOF probability) data. This conditional OOF bootstrap quantifies sampling variability given the fixed OOF predicted values and does not refit the full modeling pipeline within each bootstrap replicate.

Supplementary analysis: As a descriptive plausibility check, we performed a Firth-penalized logistic regression (Supplementary Notes; Supplementary Table S1).

## 3. Results

### 3.1. Cohort Characteristics

All 52 patients entered the analysis (0 exclusions). Secondary spinal infection occurred in 29 patients (55.8%), while primary spinal infection occurred in 23 patients (44.2%). The cohort included 29 males and 23 females, with a mean age of 60.4 years (SD 13.8). CKD/DM cross-classification showed CKD0/DM0 (*n* = 24), CKD0/DM1 (*n* = 15), CKD1/DM0 (*n* = 13), and CKD1/DM1 (*n* = 0).

Table 1 summarizes the cohort distribution and the observed risks for both secondary and primary spinal infection across supported CKD–DM strata.

The microbiological distribution of identified pathogens is summarized in Table 2.

### 3.2. Explanatory Model Performance (OOF Internal Validation)

**Figure 1:**
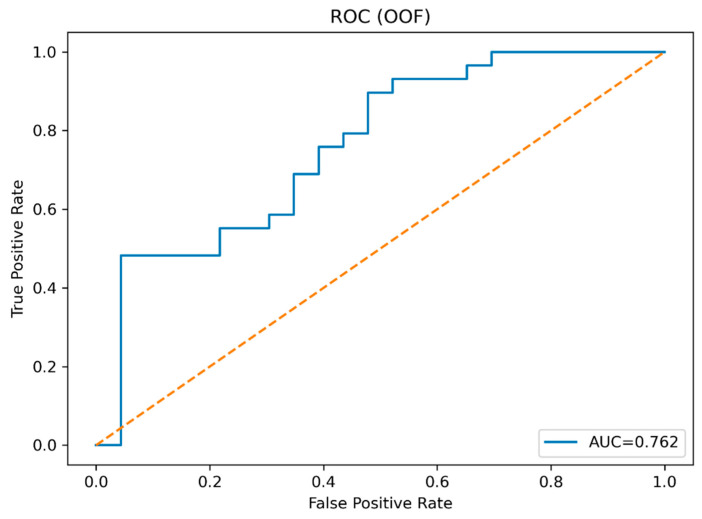
ROC curve for OOF scores (ridge model). The dashed diagonal line indicates the line of no discrimination, corresponding to random classification performance.

The ridge model, evaluated under strict internal validation with leakage precautions, demonstrated a clear discriminative signal for spinal infection probabilistic profile differentiation (OOF AUC 0.762; Table 3; Figure 1). This separation was symmetric, with opposite-direction probabilistic profile alignment: DM-only patients aligned with the secondary probabilistic profile, whereas CKD-only patients aligned with the primary probabilistic profile.

Specifically, DM-only patients (CKD0/DM1) had an observed secondary infection risk of 93.3% and a mean OOF predicted probability of 84.4%, whereas CKD-only patients (CKD1/DM0) had an observed secondary infection risk of 15.4% (implying primary risk 84.6%) and a mean OOF predicted probability of 21.8%. The CKD0/DM0 stratum provided an intermediate reference profile.

**Figure 2:**
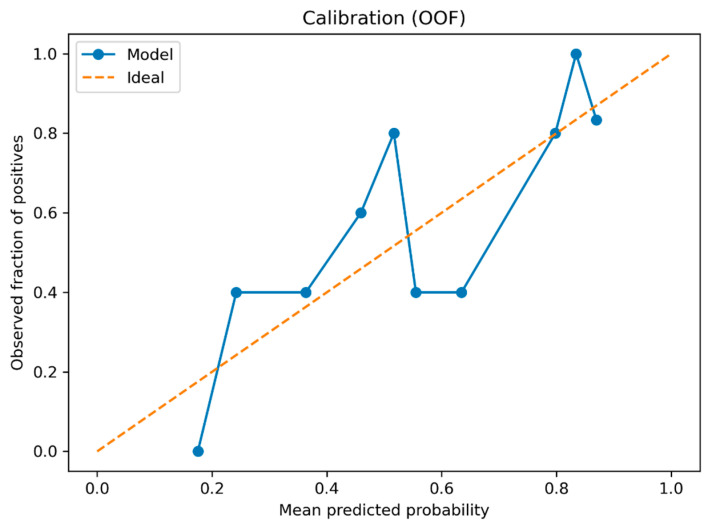
Calibration curve for single-valued patient-level OOF probabilities (quantile bins).

Calibration and overall error supported that these probabilistic profile-aligned probability assignments were not artifacts of overly extreme risk scores: calibration point estimates were near-ideal (intercept 0.069; slope 1.028), with uncertainty summarized by conditional OOF bootstrap (Table 3). The OOF calibration curve is shown in Figure 2.

### 3.3. Decision-Analytic Utility

**Figure 3:**
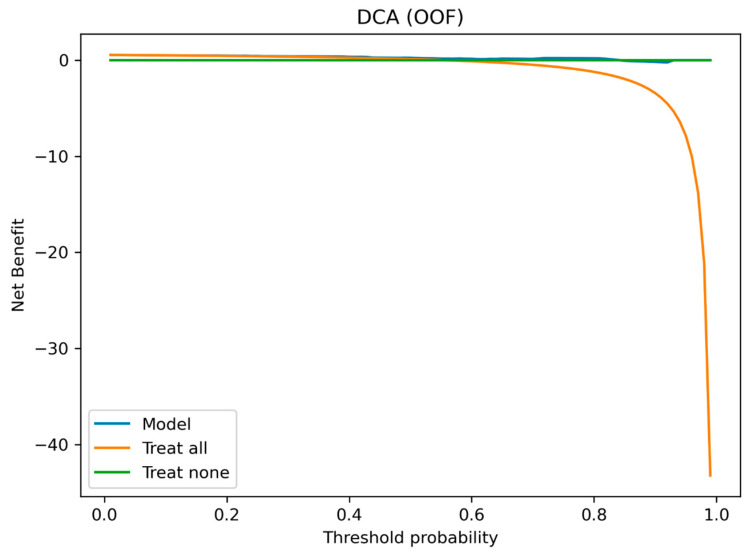
Decision curve analysis (DCA) using single-valued OOF probabilities.

Decision curve analysis (DCA) indicated positive net benefit for etiologic differentiation across a broad threshold range (approximately 0.15–0.84), supporting use of the predicted probability as a triage and diagnostic-vigilance signal across clinically plausible operating points (Figure 3).

### 3.4. Support-Aware CKD–DM Group Profiles

**Figure 4:**
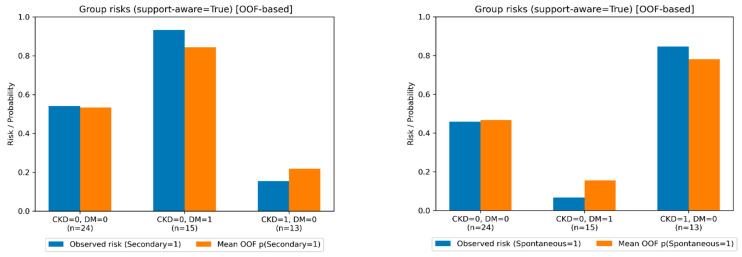
Support-aware CKD–DM subgroup profiles: observed risk vs. mean patient-level OOF predicted probability, restricted to CKD–DM strata supported by the data.

Support-aware subgroup summaries highlight probabilistic profile alignment in the supported CKD–DM strata. DM-only (CKD0/DM1) shows high observed probability of the secondary probabilistic profile, whereas CKD-only (CKD1/DM0) shows high observed probability of the s primary probabilistic profile; the mean patient-level OOF predicted probabilities closely track these group-level patterns. Figure 4 visualizes observed secondary and primary risks and mean patient-level OOF predicted probability of the secondary and primary probabilistic profile by CKD–DM stratum. The corresponding full-data fit version is provided in Supplementary Figure S1.

### 3.5. Final Ridge Model Presentation

For reproducibility and prospective implementation, we report a final ridge model refit on the full dataset (Table 4), including standardized coefficients, back-transformed coefficients, and the z-scoring parameters. This presentation model is not used for internal performance estimation; all performance metrics and plots are based on patient-level OOF probabilities from repeated nested cross-validation. To keep probabilistic profile interpretation symmetric, we also display approximate odds-ratio scaling for both the secondary probabilistic profile (coded positive) and the primary probabilistic profile (complement), as descriptive model-implied scalings. Coefficients were obtained by selecting C via 5-fold stratified cross-validation on the full dataset (same log-loss criterion and random_state = 42), then refitting the ridge model on the full dataset at the selected C.

Association-oriented plausibility results are provided in Supplementary Table S1.

## 4. Discussion

According to Nagashima et al. [15]., spinal infection rates were well-controlled until the mid-1990s; however, the incidence has increased in recent years. Additionally, the average age of patients with spinal infections has risen, and the number of immunocompromised individuals has significantly increased. The primary reasons for this trend include the rising age of disease onset due to advances in healthcare technologies and the growing prevalence of immunosuppressed patients resulting from various factors. We believe that the CKD and DM patient groups, which were prominently featured in our study, are the underlying factors contributing to immunosuppression.

Age is a significant factor influencing mortality in spinal infections. In a large cohort study on spondylodiscitis, Gerstmeyer et al. [16]. identified patients over the age of 65—particularly those over 80 years—as a high-risk group. Furthermore, the same study emphasized that early diagnosis and treatment were the most critical factors in reducing mortality. Veljanoski et al. [2]. showed a case series of patients presenting with thoracic symptoms, limb weakness and cauida equina syndrome with a poor prognosis. They observed that patients with isolated back pain and a history of malignancy had a good prognosis. In cases of primary spinal infections, factors such as the presence of an epidural abscess, involvement of the cervical or thoracic spine, and a greater number of affected spinal levels have been identified as potential predictors for requiring surgical intervention [17].

Staphylococcus aureus is the most frequently isolated pathogen in spinal infections [18,19]. However, in individuals with a history of intravenous drug use, Pseudomonas aeruginosa and Mycobacterium tuberculosis should also be considered as important causative organisms. While Chuo et al. [20]., in a retrospective study of 21 intravenous drug users, identified Staphylococcus aureus as the most common pathogen, case reports by Smith et al. [21]. and Bryan et al. [22]. have documented Pseudomonas aeruginosa as etiologic agents in this specific patient population. These findings underscore the need for tailored microbiological evaluation and empiric antimicrobial therapy in intravenous drug users presenting spinal infections. Miksić et al. pointed out that the majority of spinal infections in developed countries are caused by pyogenic bacteria, with Staphylococcus aureus among gram positive cocci and Escherichia coli among gram negative bacteria, while coagulase-negative staphylococci often play a role in implant-related spinal infections. Implant related spinal infections were caused by bacteria that can produce biofilms on the implant surface and thus become resistant to most antimicrobial drugs. While spinal infections in non-implant patients can be treated conservatively with pathogen directed antimicrobial therapy, implant related spinal infections require combined surgical and antibiotic therapy. In the era of drug resistant pathogens, empirical antimicrobial treatment of spinal infections without microbiologic diagnosis is not recommended [23]. The implant can be expected to eliminate spinal infection, but removal of the implant is often necessary. Complete removal of implants allows significant progression of the degeneration and deformity requiring the implant [24].

The application of spinal instrumentation in the setting of active infection continues to be a topic of clinical controversy. A review of the current literature indicates no statistically significant difference in the rates of recurrent infection or the need for reoperation between patients with primary spinal infections treated with decompression alone versus those managed with both decompression and instrumentation. Based on these findings, spinal instrumentation may be considered appropriate in cases where spinal stability remains compromised following surgical intervention for infection [25,26,27]. In a retrospective study of 627 patients who required surgery after primary spinal infection, preoperative hypoalbuminemia and the presence of dialysis were found to be major postoperative morbidity and mortality risks [28].

In a comparative study comparing the clinical, radiologic and laboratory characteristics of patients with spinal tuberculosis and patients with non-specific spinal infection, it was observed that individuals in the non-specific infection group were generally older and had higher body temperatures, higher leukocyte counts, erythrocyte sedimentation rates (ESR) and C-reactive protein (CRP) levels. In addition, the onset of symptoms was more sudden in this group. Radiological assessments more commonly revealed features such as osseous bridging and the presence of sequestra [29].

Parasitic infections of the spine are more frequently encountered in developing countries compared to developed nations. Within the vertebral column, the thoracic region is most commonly affected [30]. Due to their often nonspecific clinical presentation, an accurate diagnosis of parasitic spinal infections requires a combination of imaging modalities, laboratory findings indicative of parasitic etiology, and, most importantly, a detailed and comprehensive patient history [31]. Cases of primary spinal hydatid cysts associated with HIV related immunosuppression have been documented in the literature [32]. However, in the single case of spinal hydatid cyst included in our study, no other immunosuppressive condition was identified.

JC Lee et al. [13]. identified several factors contributing to culture negative results in secondary spinal infections, including the administration of antibiotics prior to obtaining tissue samples from the affected site, the presence of slow growing microorganisms, and the tendency to overlook common contaminants like Staphylococcus epidermidis, which could, in fact, be the underlying cause of postoperative infections. Additionally, he observed that culture negative cases had lower revision surgery rates compared to culture positive cases, implying that culture negative secondary infections may be associated with a favorable prognosis.

Analysis of growth patterns of secondary infections after spine surgery showed no significant difference in clinical pattern or severity between polymicrobial and monomicrobial infections [33].

The rate of secondary infections was found to be significantly higher in emergency spine surgery compared to planned surgery. Blam et al. reported that prolonged time to surgery and prolonged postoperative stay in the intensive care unit were independent risk factors contributing to the development of secondary infections in the context of acute spine trauma [34].

A review of the literature reveals that the etiology of spinal infections has been extensively discussed, and predisposing factors have been presented. CKD primarily causes spinal infections via the hematogenous pathway, while DM often leads to spinal infections due to its immunosuppressive effects in patients following spinal invasive procedures. Although CKD and DM are among these predisposing factors, early-phase exploratory modeling for primary and secondary spinal infections is lacking.

### 4.1. Principal Findings

This study shows that a rigorously validated ridge penalized approach can extract a clear discriminative signal distinguishing secondary and primary spinal infection probabilistic profiles in an early-stage cohort. Significantly, the model did not favor one etiology over another: it distinguished a DM-dominant probabilistic profile consistent with secondary spinal infection from a CKD-dominant probabilistic profile consistent with primary spinal infection, while maintaining near-ideal calibration in point estimates.

### 4.2. Probabilistic Profile Separation and Interpretation

Given that the outcome is coded as a binary opposition (secondary = 1 versus primary = 0), directional patterns should be interpreted as probabilistic profile alignment within this etiologic discrimination task, rather than mechanistic or causal effects. In this framework, DM aligns with the secondary probabilistic profile and CKD aligns with the primary probabilistic profile (complementary class) within the supported CKD-DM strata. This interpretation maintains clinical neutrality while directly addressing the two probabilistic profile questions that motivated the study.

### 4.3. Analytical Rigor and Leakage Prevention

Analytically, we emphasize leakage precautions in preprocessing, repeated nested cross-validation for hyperparameter tuning and OOF likelihood estimation, and explicit reporting of discrimination, calibration, overall error and decision analysis utility. Tuning was performed by minimizing log-loss to prioritize probability accuracy and aligning the analysis objective with probability-based clinical decision making.

### 4.4. Clinical Implications

The ability to distinguish between secondary and primary spinal infection probabilistic profiles using a small number of predictive factors may support early diagnostic triage and vigilance. In practical terms, a higher predictive probability of secondary etiology may justify earlier and more intensive resource exploration (including targeted imaging and microbiological sampling) and closer coordination of diagnostic steps, whereas a higher predictive probability of primary etiology may prioritize assessment of hematologic dissemination, risk factor assessment, and structured monitoring while diagnostic results are evolving. Decision curve analysis suggests that such etiologic differentiation may provide net benefit across a wide range of thresholds, supporting both ruling-in secondary etiology at higher thresholds and ruling-it-out in favor of primary etiology at lower thresholds, depending on local decision context.

### 4.5. Scope Boundaries and Next Steps

Reporting was restricted to CKD–DM strata supported by the dataset; the CKD+DM+ subgroup was not represented (*n* = 0) and therefore was not modeled as an estimable stratum. The present results provide reproducible internally validated performance estimates and an analysis approach that can be carried forward into subsequent external validation studies, where recalibration and threshold specification can be tailored to local practice and prevalence.

This study has several limitations. Its retrospective design and relatively small sample size may limit its generalizability. The multicenter structure includes only two institutions, which may not fully represent broader application models. Furthermore, external validation has not been performed; therefore, the findings should be interpreted as hypothesis-generating and should be validated in larger, prospective cohorts and we plan to conduct external validation once an adequately powered independent dataset becomes available. Because key determinants of secondary infection (e.g., recent surgery/instrumentation, bacteremia source, dialysis access/vascular devices, and immunosuppressive therapy) were not captured, CKD and DM may partly act as proxies for unmeasured healthcare exposure; therefore, causal interpretations should be avoided. Moreover, since the secondary label is partly anchored to prior procedures/healthcare context, the model may primarily separate healthcare-associated (procedure-related) versus hematogenous/primary contexts rather than biological probabilistic profiles. Accordingly, all reported associations are non-causal and may reflect residual confounding. The absence of patients with both CKD and DM is a major limitation. Thus, any model-based probability assignments for this subgroup would be out-of-distribution and should be interpreted with caution. Interaction terms were not modelled due to zero empirical support. Given the limited sample size and events-per-predictor, estimates may be unstable and should not be used for clinical decision-making.

## 5. Conclusions

In this study, we differentiated primary and secondary spinal infection probabilistic profiles and evaluated the associations of CKD and DM using an internally validated ridge-penalized logistic regression model. CKD was found to be aligned with probabilistic primary spinal infections, whereas DM was aligned with probabilistic profile secondary spinal infections.

These probabilistic profile-aligned probabilities are intended to support early diagnostic triage rather than treatment decisions. A higher probability of secondary infection may prompt earlier investigation for an identifiable source and closer coordination of diagnostic steps, while a higher probability of primary infection may prioritize evaluation for hematogenous spread and structured clinical monitoring.

Although internally validated using resampling-based methods, these findings require confirmation in larger, multicenter cohorts. Future studies should focus on external validation, inclusion of broader patient strata, and assessment of clinical utility across different institutional settings. Time from symptom onset to diagnosis was not available in a structured form and could not be analyzed; future prospective cohorts should capture diagnostic delay given its potential influence on infection phenotype.

## Figures and Tables

**Table 1:** Cohort and CKD/DM subgroup distribution.

Group	Total (*n*)	Secondary Infection (*n*)	Observed Risk (Secondary = 1)	Observed Risk (Primary = 0)
Overall	52	29	0.558	0.442
CKD = 0, DM = 0	24	13	0.542	0.458
CKD = 0, DM = 1	15	14	0.933	0.067
CKD = 1, DM = 0	13	2	0.154	0.846

**Table 2:** Distribution of Identified Pathogens.

Pathogen	*n*	%
No growth	19	36.5
Klebsiella	3	5.8
Sphingomonas	1	1.9
Mycobacterium tuberculosis	8	15.4
Brucella	1	1.9
Staphylococcus haemolyticus	3	5.8
Staphylococcus aureus	7	13.5
Staphylococcus epidermidis	3	5.8
Pseudomonas	3	5.8
Acinetobacter	2	3.8
Echinococcus	1	1.9
Enterococcus	1	1.9

**Table 3:** OOF performance with conditional OOF bootstrap uncertainty.

Metric	Point Estimate OOF	Median	Ci Low 2.5	Ci High 97.5
AUC	0.762	0.765	0.608	0.885
Brier	0.189	0.188	0.141	0.243
Log Loss	0.559	0.556	0.445	0.694
Cal Intercept	0.069	0.077	−0.590	0.761
Cal Slope	1.028	1.076	0.457	1.961

**Table 4:** Final ridge model presentation.

Term	Coef (Standardized Scale)	Coef (Original Units)	Approx OR (Secondary = 1 vs. Primary = 0)	Mean Used for Z-Scoring	SD Used for Z-Scoring	Reciprocal OR (Primary vs. Secondary)
Intercept	0.246	−1.234	0.291			
Sex	−0.003	−0.007	0.993	0.442	0.497	1.007
Age	0.323	0.024	1.024	60.385	13.701	0.977
Ckd	−0.662	−1.529	0.217	0.250	0.433	4.608
Dm	0.696	1.536	4.646	0.288	0.453	0.215

Approximate ORs are exponentiated ridge-penalized coefficients on the original-unit scale (back-transformed from the standardized fit) and are reported descriptively (no CIs). Outcome coding: Secondary = 1 vs. Primary = 0; therefore OR > 1 indicates higher odds of Secondary infection. Inferential ORs and 95% CIs are reported separately using adjusted Firth logistic regression (age per 1-year increase; binary predictors 1 vs. 0; CKD0–DM0 as reference). Reciprocal OR is shown for convenience (1/OR).

## Data Availability

The original contributions presented in this study are included in the article. Further inquiries can be directed to the corresponding author.

## Supplementary Materials

The following supporting information can be downloaded at: https://www.mdpi.com/article/10.3390/jcm15051873/s1, Figure S1: Support-aware CKD–DM subgroup profiles (full-data fit): observed risk vs. model-implied mean probability; Table S1: Firth-penalized association summary.

## Abbreviations

The following abbreviations are used in this manuscript:

CKD	Chronic kidney disease
DM	Diabetes mellitus
CV	Cross-validation
OOF	Out-of-fold
AUC	Area under the curve
mNGS	Metagenomic next generation sequencing
