# Supplementary material for: Secondary vs. Primary Spinal Infection in Early Clinical Assessment: A Parsimonious, Leakage-Resistant Modelling Approach with Internal Validation: A Multicenter Retrospective Study
Source: J Clin Med. 2026 Feb 28;15(5):1873. doi: 10.3390/jcm15051873 (PMC12986406; doi:10.3390/jcm15051873)
Supplement: Supplementary file 1 [file jcm-15-01873-s001.zip › jcm-4133971-supplementary.pdf]

Supplementary Notes

- Model (plausibility track): Firth-penalized logistic regression adjusted for sex, age, and CKD/DM group contrasts (association-oriented plausibility check; descriptive, non-causal).
- Reference group: CKD0_DM0 (neither CKD nor DM).
- CKD1_DM1 (CKD+DM+): Absent in the dataset (n=0) and therefore not estimable; not reported.
- Age (per 1 year): Mean-centered for numerical stability; centering mean was 60.384615 (meta sheet).
- Meta: n_used=52; converged=True; n_iter=9; positive class = infectiontype=1 (Secondary).
- Random seed: 42 (used for outer and inner cross-validation shuffling/repeats).
- Outer validation: RepeatedStratifiedKFold with 5 splits and 10 repeats.
- Inner tuning: StratifiedKFold with 5 splits, shuffle=True.
- Ridge grid: C in {1e-4, ..., 1e4} with 21 log-spaced values (logspace(-4, 4, 21)).
- Optimizer: liblinear solver with max_iter=5000.
- Calibration plot: 10 quantile bins.
- Decision curve analysis: thresholds 0.01 to 0.99 (99 points).
- Final coefficient table: C selected by 5-fold CV on full data then refit on full data at the selected C.
- Firth plausibility check: adjusted-score iterations (max_iter=200; tol=1e-8) with Wald-type intervals.

Group-risk visualizations and subgroup summaries were restricted to clinically meaningful CKD–DM strata with adequate support. Sex and age were retained as covariates in the prediction model; subgroup visualization by sex was avoided to prevent unstable cell counts, preserving focus on the primary etiologic discrimination task.

Supplementary association summary (Firth; plausibility check)

Firth-penalized regression provided an association-oriented plausibility check that mirrored the phenotype alignment observed in the prediction model. DM-only (CKD0/DM1 vs CKD0/DM0) aligned with the secondary spinal infection phenotype (adjusted OR for secondary = 6.36). For CKD-only (CKD1/DM0 vs CKD0/DM0), the fitted contrast under the secondary-coded model corresponds to alignment with the spontaneous phenotype; equivalently, the odds of spontaneous (vs secondary) spinal infection were approximately 5.00 (i.e., 1/0.20), with the corresponding interval obtained by reciprocation of the secondary-coded estimate.

Supplementary Table S1. Firth-penalized association summary

Predictor / Contrast	β (Firth)	SE	95% CI (β)	Adjusted OR	95% CI (OR)
Intercept (log-odds; at reference group)	0.194	0.502	−0.789 to 1.178	—	—
Sex (Female vs Male)	0.032	0.676	−1.292 to 1.357	1.033	0.275 to 3.883
Age (per 1 year) *	0.023	0.025	−0.026 to 0.072	1.023	0.974 to 1.075
CKD0/DM1 vs CKD0/DM0 (DM only vs none)	1.849	0.991	−0.092 to 3.791	6.356	0.912 to 44.305
CKD1/DM0 vs CKD0/DM0 (CKD only vs none)	−1.610	0.830	−3.236 to 0.016	0.200	0.039 to 1.016

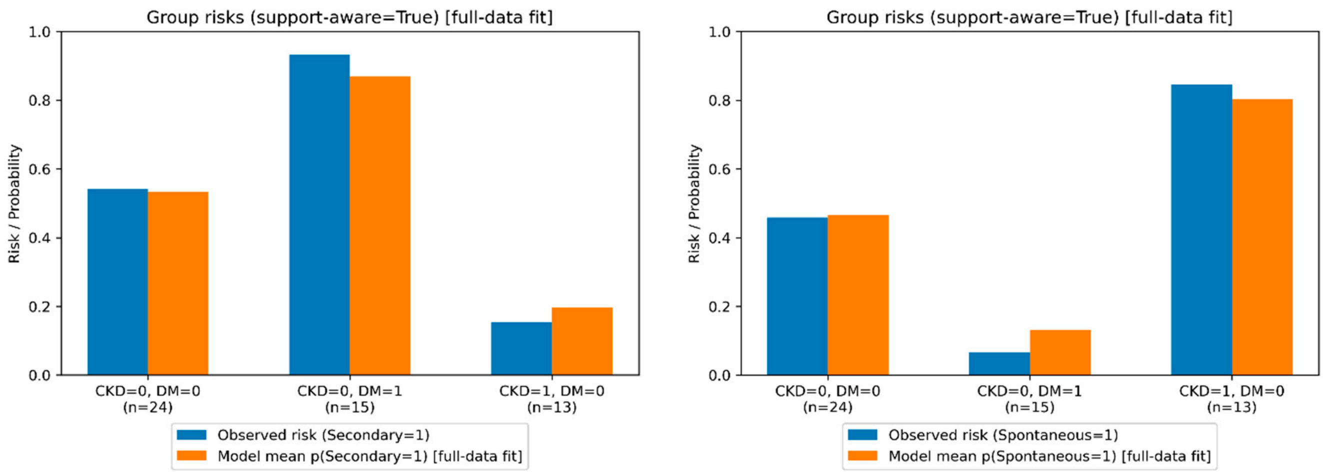

Supplementary Figure 1. Support-aware CKD–DM subgroup profiles (full-data fit): observed risk vs model-implied mean probability
